# MP29-02*’s advanced delivery system contributes to its efficacy in patients with moderate/severe seasonal allergic rhinitis

**DOI:** 10.1186/2045-7022-5-S4-P36

**Published:** 2015-06-26

**Authors:** Glenis Scadding, Claus Bachert, Peter Hellings, Wytske Fokkens, Ullrich Munzel, Ralph Mösges

**Affiliations:** 1 The Royal National Throat Nose and Ear Hospital, London, UK; 2 Ghent University Hospital, Department of Oto-Rhinolaryngology, Ghent, Belgium; 3 University Hospitals Leuven, Dept of Otorhinolaryngology, Head & Neck Surgery, Leuven, Belgium; 4 Academic Medical Center, Department of Otorhinolaryngology, Amsterdam, Netherlands; 5 Meda, Corporate Clinical Affairs, Bad Homburg, Germany; 6 University of Cologne, IMSIE, Cologne, Germany

## Background

Four previously published trials assessed the efficacy of MP29-02* (a novel intranasal formulation of azelastine hydrochloride (AZE) and fluticasone propionate (FP) in an advanced delivery system) in seasonal allergic rhinitis (SAR) [1,2]. The first study compared MP29-02* to marketed AZE and FP [2]. The others compared MP29-02* to AZE and FP in the MP29-02* vehicle and delivery device (i.e. re-formulated comparators) [1]. FP contained within MP29-02* has a unique PK fingerprint [3]. The aim of this analysis was to demonstrate that formulation/device contribute to MP29-02*’s clinical efficacy.

## Methods

Four thousand and five moderate/severe SAR patients (≥12 yrs old) were randomized into 4 double-blind, placebo (PLA)-controlled trials. Each trial comprised 4 groups: MP29-02*, AZE, FP and PLA nasal sprays, and was conducted for 14 days. Total daily dose of AZE and FP were 548 µg and 200 µg, respectively. Change from baseline (CFB) in reflective total nasal symptom score (rTNSS) over 14-days was the primary outcome. CFB in reflective total ocular symptom score (rTOSS) and individual nasal and ocular symptoms was assessed secondarily. Time to achieve at least a 50% rTNSS reduction from baseline was assessed post-hoc by Kaplan Meier estimates and log rank tests. The formulation/device effect of MP29-02* was quantified by comparing treatment differences obtained with MP29-02* vs marketed FP and MP29-02* vs re-formulated FP for these endpoints.

## Results

For all efficacy variables assessed, the treatment difference was greater for MP29-02* vs marketed-FP than for MP29-02* vs re-formulated-FP. For rTNSS, the difference between MP29-02* and marketed-FP was -1.47, compared to -0.76 vs reformulated-FP; a formulation/device effect of 0.71. Similarly for rTOSS a formulation/device effect of 0.70 was observed. A formulation/device effect was observed for relief of all individual nasal and ocular symptoms (e.g. 0.23 effect for congestion; 0.34 effect for ocular itching). Finally, MP29-02*-patients achieved a ≥50% rTNSS reduction ≤6 days faster than marketed-FP and ≤3 days faster than reformulated-FP, a formulation/device effect of ≤3 days.

## Conclusion

Formulation and device contribute to MP29-02*’s superior efficacy over currently considered firstline therapy, making MP29-02* a new class of treatment for AR.

*Dymista
